# Prognostic value of immunotherapy-induced organ inflammation assessed on ^18^FDG PET in patients with metastatic non-small cell lung cancer

**DOI:** 10.1007/s00259-022-05788-8

**Published:** 2022-05-14

**Authors:** Olivier Humbert, Matteo Bauckneht, Jocelyn Gal, Marie Paquet, David Chardin, David Rener, Aurelie Schiazza, Carlo Genova, Renaud Schiappa, Lodovica Zullo, Giovanni Rossi, Nicolas Martin, Florent Hugonnet, Jacques Darcourt, Silvia Morbelli, Josiane Otto

**Affiliations:** 1 Department of Nuclear Medicine, Centre Antoine-Lacassagne, Université Côte d’Azur (UCA), 33 Avenue de Valombrose, 06189 Nice, France; 2 TIRO-UMR E 4320, UCA/CEA, Nice, France; 3 Nuclear Medicine Unit, IRCCS Ospedale Policlinico San Martino, Genoa, Italy; 4 Department of Health Sciences, University of Genoa, Genoa, Italy; 5 Department of Biostatistics, Centre Antoine-Lacassagne, Nice, France; 6 UOC Clinica Di Oncologia Medica, IRCCS Ospedale Policlinico San Martino, Genoa, Italy; 7 Dipartimento Di Medicina Interna E Specialità Mediche (DiMI), Facoltà Di Medicina E Chirurgia, Università Degli Studi Di Genova, Genoa, Italy; 8 Department of Medical, Surgical and Experimental Sciences, University of Sassari, Sassari, Italy; 9 UO Oncologia Medica, Ospedale Padre Antero Micone, Genoa, Italy; 10 Department of Medical Oncology, Centre Antoine-Lacassagne, UCA, Nice, France; 11 Department of Nuclear Medicine, Centre Hospitalier Princesse Grâce, Monaco, Monaco

**Keywords:** FDG PET, Immunotherapy, Lung cancer, Adverse events, Biomarker

## Abstract

**Purpose:**

We evaluated the prognostic value of immunotherapy-induced organ inflammation observed on ^18^FDG PET in patients with non-small cell lung cancer (NSCLC) treated with immune checkpoint inhibitors (ICPIs).

**Methods:**

Data from patients with IIIB/IV NSCLC included in two different prospective trials were analyzed. ^18^FDG PET/CT exams were performed at baseline (PET_Baseline_) and repeated after 7–8 weeks (PET_Interim_1) and 12–16 weeks (PET_Interim_2) of treatment, using iPERCIST for tumor response evaluation. The occurrence of abnormal organ ^18^FDG uptake, deemed to be due to ICPI-related organ inflammation, was collected.

**Results:**

Exploratory cohort (Nice, France): PET_Interim_1 and PET_Interim_2 revealed the occurrence of at least one ICPI-induced organ inflammation in 72.8% of patients, including midgut/hindgut inflammation (33.7%), gastritis (21.7%), thyroiditis (18.5%), pneumonitis (17.4%), and other organ inflammations (9.8%). iPERCIST tumor response was associated with improved progression-free survival (*p* < 0.001). iPERCIST tumor response and immuno-induced gastritis assessed on PET were both associated with improved overall survival (OS) (*p* < 0.001 and *p* = 0.032). Combining these two independent variables, we built a model predicting patients’ 2-year OS with a sensitivity of 80.3% and a specificity of 69.2% (AUC = 72.7). Validation cohort (Genova, Italy): Immuno-induced gastritis (19.6% of patients) was associated with improved OS (*p* = 0.04). The model built previously predicted 2-year OS with a sensitivity and specificity of 72.0% and 63.6% (AUC = 70.7) and 3-year OS with a sensitivity and specificity of 69.2% and 80.0% (AUC = 78.2).

**Conclusion:**

Immuno-induced gastritis revealed by early interim ^18^FDG PET in around 20% of patients with NSCLC treated with ICPI is a novel and reproducible imaging biomarker of improved OS.

**Supplementary Information:**

The online version contains supplementary material available at 10.1007/s00259-022-05788-8.

## Introduction

The recent use of immune checkpoint inhibitors (ICPIs) in metastatic non-small cell lung cancer (NSCLC) has demonstrated high improvement in patients’ outcomes and has become a standard of care in the first-line setting of metastatic NSCLC for patients without oncogenic driver mutations [1, 2]. Nonetheless, some caveats remain concerning predictive and prognostic biomarkers to guide the therapeutic choices. High PD-L1 expression is associated with better tumor response to ICPIs [3]. However, a subset of patients with low/no PD-L1 expression unexpectedly respond to ICPIs, and some patients with high PD-L1 expression do not. Therefore, predicting patient response to ICPIs early on is a growing area of research and several new promising biomarkers of response are currently under investigation [4]. Meanwhile, ICPIs are currently used in combination with chemotherapy for most patients.

By activating the immune system, ICPIs can lead to various inflammatory adverse reactions, termed immune-related adverse events (irAEs) [5, 6]. A variety of organ inflammatory impairments can occur [7, 8]. Although no widely validated strategy for the detection and follow-up of irAEs is available, it is admitted that early identification and management of these irAEs is a key issue to achieve the best patient care, such as steroid treatments initiation (≥ grade II) [9, 10]. Some studies have also suggested that symptomatic irAEs may be associated with a more favorable outcome to PD-1 inhibitors in patients with melanoma or NSCLC [9, 11].

Due to its high sensitivity for lesion detection, fluorine-18-fluorodeoxyglucose (^18^FDG) positron emission tomography (PET)/computed tomography (CT) is recommended for the pre-therapeutic staging of NSCLC [12, 13]. We and others have also shown that PET/CT is also relevant for the monitoring of NSCLC response to ICPI [14–16]. Because inflammatory tissues also have a high glucose avidity [17], immune-related inflammatory findings are frequently detected by ^18^FDG PET performed in the setting of ICPI response evaluation. Even if they do not necessarily cause symptoms, these ICPI-induced organ inflammations are usually reported for the oncologist to ensure close clinical monitoring and, in some patients, therapeutic management of irAEs.

The aim of this study was to investigate the occurrence and prognostic value of pathological organ ^18^FDG uptake related to organ inflammation to ICPI in patients with metastatic lung cancer.

## Patients and methods

### Population

To identify and externally validate ^18^FDG PET-CT predictive and prognostic biomarkers in patients with NSCLC treated with ICPIs, we analyzed data of two independent cohorts prospectively started in this setting in Nice (France) and Genova (Italy). Both studies were approved by the concerned ethics committee and regulatory agencies and informed consent was obtained from all included patients (Genova cohort: NCT02475382; Nice cohort: n°ID-RCB:2018-A02116-49/ID-RCB:2018-A00915-50). The present retrospective ancillary study was approved by the French INDS (National Health Data Institute): MR 2,610,080,620.

#### Exploratory cohort: Nice, France

From February 2017 to April 2020, 112 consecutive patients scheduled to initiate anti-PD1/PD-L1 therapy as their first or later systemic treatment for metastatic NSCLC were prospectively evaluated in an open, uncontrolled, and non-randomized current-care study in Centre Antoine Lacassagne, Nice. The inclusion criteria were as follows: (1) pathologically proven stage IIIB or IV NSCLC, irrespective of the histologic subtype; (2) an indication to start ICPI in monotherapy and in first or later line; (3) ECOG performance status of 0 to 2; (4) age of at least 18 years. The exclusion criteria were as follows: (1) clinical or biological contraindication for immune checkpoint inhibitors; (2) evidence of concurrent cancer; (3) vulnerable patients as defined in Article L1121-5 to -8 of the French Public Health Code; (4) refusal of written consent; (5) high glycemia at baseline ^18^FDG PET scan (> 9 mmol/l); (6) no measurable lesion by PERCIST V1.0.

Patients received one of three possible treatment regimens: either pembrolizumab administered intravenously at a standard dose of 2 mg/kg or 200 mg every 3 weeks, nivolumab at a standard dose of 240 mg every 2 weeks, or atezolizumab at a standard dose of 1200 mg every 3 weeks. ^18^FDG PET scans were performed within 2 months before the start of ICPIs (PET_Baseline_), after 7 weeks (PET_Interim_1), and after 3 months (PET_Interim_2) of treatment.

In case of progressive disease on PET_Interim_1, the clinical status of the patients was considered. If no clinical worsening was observed, the treatment was continued until PET_Interim_2, based on the previously described immune-related atypical response patterns [14–16, 18].

Two different PET/CT imaging systems were used: a Biograph mCT PET/CT scanner from February 2017 to September 2019 and a Biograph Vision 600 PET/CT scanner from September 2019 to April 2020 (Siemens Healthcare, Erlangen, Germany). Both PET systems fulfilled the EARL accreditation specifications for FDG PET/CT tumor imaging. Patients were instructed to fast for at least 6 h before the intravenous injection of 3 MBq/kg (Biograph mCT PET/CT) or 2.5 MBq/kg (Biograph Vision 600 PET/CT) of ^18^FDG. A low-dose attenuation CT acquisition (80 kV, 50 mA, 5 mm slice thickness) was performed 60 ± 5 min after the administration of ^18^FDG, followed by an inspiratory chest-restricted diagnostic CT (auto-kV, auto-mA, 1-mm-slice thickness). Lastly, a diagnostic CT acquisition was done from the vertex of skull to mid-thigh (auto-kV, auto-mA, 1-mm-slice thickness) after a venous injection of iodinated contrast agent in the absence of allergy or renal impairment. The same imaging system and the same acquisition parameters (duration and delay from injection) were used for baseline and post-treatment studies.

#### Validation cohort: Genova, Italy

From May 2015 to April 2016, 49 patients with advanced pre-treated NSCLC were prospectively enrolled in a translational research trial at the Lung Cancer Unit of the IRCCS Policlinico San Martino. The trial was an ancillary single-institution study conducted within the expanded-access program for nivolumab (NCT02475382). Accordingly, the specific study design was approved by the research committee of Regione Liguria.

The inclusion criteria were as follows: (1) histologically or cytologically confirmed NSCLC; (2) a clinical stage of IIIB or IV (TNM, version 7.0); (3) at least one previous line of therapy; (4) at least one measurable lesion by RECIST 1.1; (5) ECOG performance status 0 to 2; (6) age of at least 18 years. The exclusion criteria were (1) meningeal carcinomatosis; (2) active autoimmune disease or a syndrome requiring daily steroid treatment; (3) a previous line of therapy with ICPIs; (4) the administration of a live attenuated vaccine within the 30 days before the first nivolumab administration; (5) untreated brain metastases or brain metastase(s) proven to be evolutive less than 2 weeks before nivolumab initiation; and (6) high glycaemia at baseline ^18^FDG PET scan (> 160 mg/dL).

Patients received nivolumab at a standard dose of 3 mg/kg every 2 weeks. ^18^FDG PET scans were performed within 30 days before the start of nivolumab (PET_Baseline_) and repeated after 8 weeks (i.e., 4 cycles) of treatment (PET_Interim_1). If the patient did not demonstrate a clinical worsening, treatment was continued and a subsequent PET/CT evaluation (PET_Interim_2) was performed after 2 additional cycles (i.e., 12 weeks of treatment) for patients with a progressive disease on PET_Interim_1, or after 4 additional cycles (i.e., 16 weeks of treatment) for patients with stable disease or partial response on PET_Interim_1.

A Biograph 16 PET/CT imaging system was used for all patients (Siemens Healthcare). Patients were instructed to fast overnight before the intravenous injection of 300–400 MBq of ^18^FDG. The PET/CT fulfilled the EARL accreditation program. No cross-validation or comparison of the recovery coefficient with the PET/CT of Nice was performed for this study.

### PET interpretation (Nice and Genova cohorts)

Peak standardized uptake values, normalized by body weight (SUV_peak_), were calculated in regions of interest placed on the site of the tumors or organ inflammations.

#### ***PET***_***Interim***_***1 (after 7–8 weeks of treatment)***

- The PERCIST criteria [19] but also the iPERCIST criteria, adapted to the issue of immunotherapy and inspired from previous guidelines and studies [14–16, 18], were used for the interpretation of the interim PET scans (see Supplementary Table S1 for iPERCIST criteria and definitions). At this step, the only difference between PERCIST and iPERCIST criteria is that progressive metabolic disease (PMD) is termed unconfirmed progressive metabolic disease (uPMD).
- The occurrence of pathological organ ^18^FDG uptake related to inflammation was visually and semi-quantitatively assessed by two senior nuclear physicians of Nice, France. As there is no current standard definition for the assessment of ICPI-induced organ inflammation on ^18^FDG PET, we defined it as the visual occurrence of a diffuse and homogeneous increase of the intensity of an organ uptake, not present on the PET_Baseline_. If a diffuse organ uptake was already present at baseline, it was not considered to be secondary to ICPI-related inflammation on the following PET exams, except if an increase in the extent of organ uptake was visually observed, or an increase of the intensity of the organ uptake was quantitatively assessed compared to the PET_Baseline_ (defined as a SUV_peak_ increase greater than 30%: i.e., ΔSUV_peak_ ≥ 30%). These diffuse inflammatory organ uptakes, deemed to be induced by ICPI, were collected separately for each organ (thyroiditis, gastritis, colitis, pneumonitis, hepatitis, cutaneous inflammation, pancreatitis, etc.).

#### ***PET***_***Interim***_***2 (after 12–16 weeks of treatment)***

- The iPERCIST criteria were used for the interpretation of the PET scans (Supplementary Table S1).
- As for PET_Interim_1, the occurrence of pathological ^18^FDG uptake deemed to be due to the ICPI-induced organ inflammation or immune activation was visually and semi-quantitatively assessed, using the definition previously mentioned.

### Follow-up and clinical endpoints

Patients were followed with regular clinical evaluations and standard of care imaging (including follow-up brain MRI, ^18^FDG PET, and CT exams).

Overall survival (OS) was the primary endpoint of the study. OS was recorded and defined as the time from initiation of ICPI to death from any cause.

Progression-free survival (PFS) was the second endpoint of the study and defined as the time from the initiation of ICPI to confirmed tumor progression or death. Tumor progression needed to be confirmed by a multi-disciplinary tumor board, confronting the patient’s clinical status, PET/CT results, and brain MRI results. Confirmed tumor progression necessarily implied the decision to definitively stop the treatment. For PFS, we did not take the time of first evidence of tumor progression on PET/CT due pseudo-progression or dissociated response patterns [14].

### Statistical analyses

Categorical data are shown as counts and percentages, and continuous variables as minimum, median, maximum, and means with standard deviations. Available case analysis was the approach used for missing data. OS and PFS were evaluated using the Kaplan–Meier method and estimated with 95% confidence interval (95%CI). Median follow-up with a 95% confidence interval was calculated with the reverse Kaplan–Meier method. The log-rank test was used for the univariate analysis of PFS and OS. For OS, a predictive model was built, using a multivariable Cox regression method by following a step-by-step descending variable selection procedure using the AIC criterion. The proportional hazard assumption was checked using statistical tests and graphical diagnostics based on the scaled Schoenfeld residuals [20]. A classifier predicting the risk of progression or death was based on the linear predictor given by the model. The ability of the model to predict OS at *t* = 24 months was evaluated using the area under the ROC curve (AUC). Risk groups for predictive models were obtained using the threshold value to obtain the best compromise between sensitivity and specificity. A *p* value of < 0.05 was considered statistically significant and all tests were two-sided. All statistical analyses were performed with R.3.5.2 software on Windows® and the survMisc, timeROC, and Survminer packages.

## Results

### Exploratory cohort (Nice, France)

#### ***Patients’ characteristics (Table ******1******)***

**Table 1:** Patients’ characteristics

^Characteristics^	^Exploratory cohort, Nice^	^Validation cohort, Genova^
^Number of patients, *n* (%)^	92 (100.0)	45 (100.0)
Age (years), mean ± SD	65.3 ± 10.0	68.2 ± 9.6
Sex, *n* (%)		
Men	59 (64.1)	30 (66.7)
Women	33 (35.9)	15 (33.3)
ECOG performance status, *n* (%)		
0	24 (26.1)	20 (44.4)
1	58 (63.0)	22 (48.9)
2	10 (10.9)	3 (6.7)
Tumor histology, *n* (%)		
Adenocarcinoma	72 (78.3)	33 (76.7)
Squamous cell carcinoma	16 (17.4)	10 (23.3)
Carcinoma NOS	4 (4.3)	0 (0.0)
Unknown	0	2
Current or former smoker, *n* (%)		
Yes	55 (73.3)	38 (84.4)
No	18 (24.7)	7 (15.6)
Unknown	19	0 (0.0)
Previous lung surgery, *n* (%)		
Yes	20 (21.7)	18 (40.9)
No	72 (78.3)	26 (59.1)
Unknown		1
Number of previous chemotherapy lines, *n* (%)		
None	24 (26.1)	0 (0.0)
1	34 (37.0)	15 (33.3)
2	20 (21.7)	15 (33.3)
3 or more	14 (15.2)	15 (33.3)
Previous lung radiotherapy, *n* (%)		
Yes	35 (38.0)	5 (11.1)
No	57 (62.0)	40 (88.9)
Median time (months) between previous lung radiotherapy and start of ICPI	10.7 [1–38.2]	17.1 [3.7–50.2]
PD-L1 tumor expression (%)		
< 1%	9 (12.5)	NK
1–49%	21 (29.2)	NK
≥ 50%	42 (58.3)	NK
Unknown	20	45
Treatment, *n* (%)		
Pembrolizumab	50 (54.3)	0 (0.0)
Nivolumab	39 (42.4)	45 (100.0)
Atezolizumab	3 (3.3)	0 (0.0)
iPERCIST response on PET_Interim_1, *n* (%)		
CMR	8 (8.7)	1 (2.3)
PMR	19 (20.6)	11 (25.0)
SMD	6 (6.6)	1 (2.3)
uPMD	59 (64.1)	32 (71.1)
iPERCIST response on PET_Interim_2, *n* (%)		
CMR	11 (14.7) (including 2 patients with pseudo-progression)	5 (15.6)
PMR	22 (29.3) (including 11 patients with pseudo-progression)	5 (15.6)
SMD	4 (5.3)	4 (12.5)
uPMD	10 (13.3)	2 (6.2)
cPMD	28 (37.3)	16 (40.6)
Unknown (exam waived due to early progression and treatment stop)	17	13
Median progression-free survival (months)	14.3 (IC95%: 7.1–22.7)	5.9 [IC95%: 3.2–8.7]
Median overall survival (months)	21.8 (IC95%: 16.4–NA)	9.9 (IC95%: 9.1–17.7)

*ECOG*, Eastern Cooperative Oncology Group; *NOS*, not otherwise specified; *PD-L1*, programmed death ligand 1; *CMR*, complete metabolic response; *PMR*, partial metabolic response; *SMD*, stable metabolic disease; *PMD*, progressive metabolic disease; *uPMD*, unconfirmed progressive metabolic disease; *cPMD*, confirmed progressive metabolic disease

One hundred and twelve patients were prospectively evaluated. Six patients were excluded because ICPI was finally not initiated. Ten patients were excluded because the treatment was stopped in the very first weeks of treatment, with PET_Interim_1 being waved, due to hyper-progression (*n* = 9) or severe treatment toxicity (*n* = 1). Four patients were excluded for other reasons: no target lesion on PET (*n* = 1), PET_Interim_1 cancelation, or wrong timing (*n* = 3).

The ninety-two remaining patients could therefore be included in this study. Mean patient age was 65.3 ± 10.0 years. All patients had a locally advanced or metastatic NSCLC, with the pathological type being a squamous cell carcinoma in 17.4% (16/92), adenocarcinoma in 78.3% (72/92), and carcinoma “not otherwise specified” (NOS) in 4.3% (4/92) of patients. 54.3% (50/92) of patients were treated with pembrolizumab, mostly in first line of the metastatic setting, and 42.4% (39/92) of patients were treated with nivolumab, mostly in second line. The 3.3% (3/92) of remaining patients were treated with atezolizumab.

Mean time between baseline PET and introduction of immunotherapy was 15.1 ± 16.1 days. Mean time between introduction of immunotherapy and PET_Interim_1 was 49.1 ± 6.1 days. Mean time between introduction of immunotherapy and PET_Interim_2 was 97.3 ± 14.4 days.

Patients’ median follow-up time was 25.3 months (CI 95%: 20.5–28.3). Patients’ median PFS was 14.3 months (CI 95%: 7.1–22.7) (Supplementary Fig. S1). Patients’ median OS was 21.8 months (CI 95%: 16.4–NA) (Supplementary Fig. S2).

#### ***Results of PET***_***Interim***_***1 and PET***_***Interim***_***2 exams***

The PET_Interim_1 was available for all patients. The PET_Interim_2 was available for 75 of the 92 patients as it was waived due to major clinical worsening and early treatment discontinuation in 17 patients.

Tumor response on PET_Interim_1: (i)PERCIST criteria (Table 1):

- 29.3% of patients had a metabolic complete or partial response (*n* = 27/92).
- 6.6% of patients had a stable metabolic disease (*n* = 6/92).
- 64.1% of patients had an (unconfirmed) metabolic progressive disease (*n* = 59/92).

Tumor response on PET_Interim_2: iPERCIST criteria (Table 1):

- 44.0% (33/75) of patients had a complete or partial metabolic response, 13 of them having initially shown a pseudo-progression on PET_Interim_1.
- 5.3% (4/75) of patients had a stable metabolic disease.
- 13.3% (10/75) of patients had a first, unconfirmed, progressive metabolic disease.
- 37.3% (28/75) of patients had a confirmed metabolic progressive disease (cPD), corresponding to patients with 2 consecutive PERCIST progressive diseases (including 11/28 patients with dissociated evolution of lesions).

**Fig. 1:**
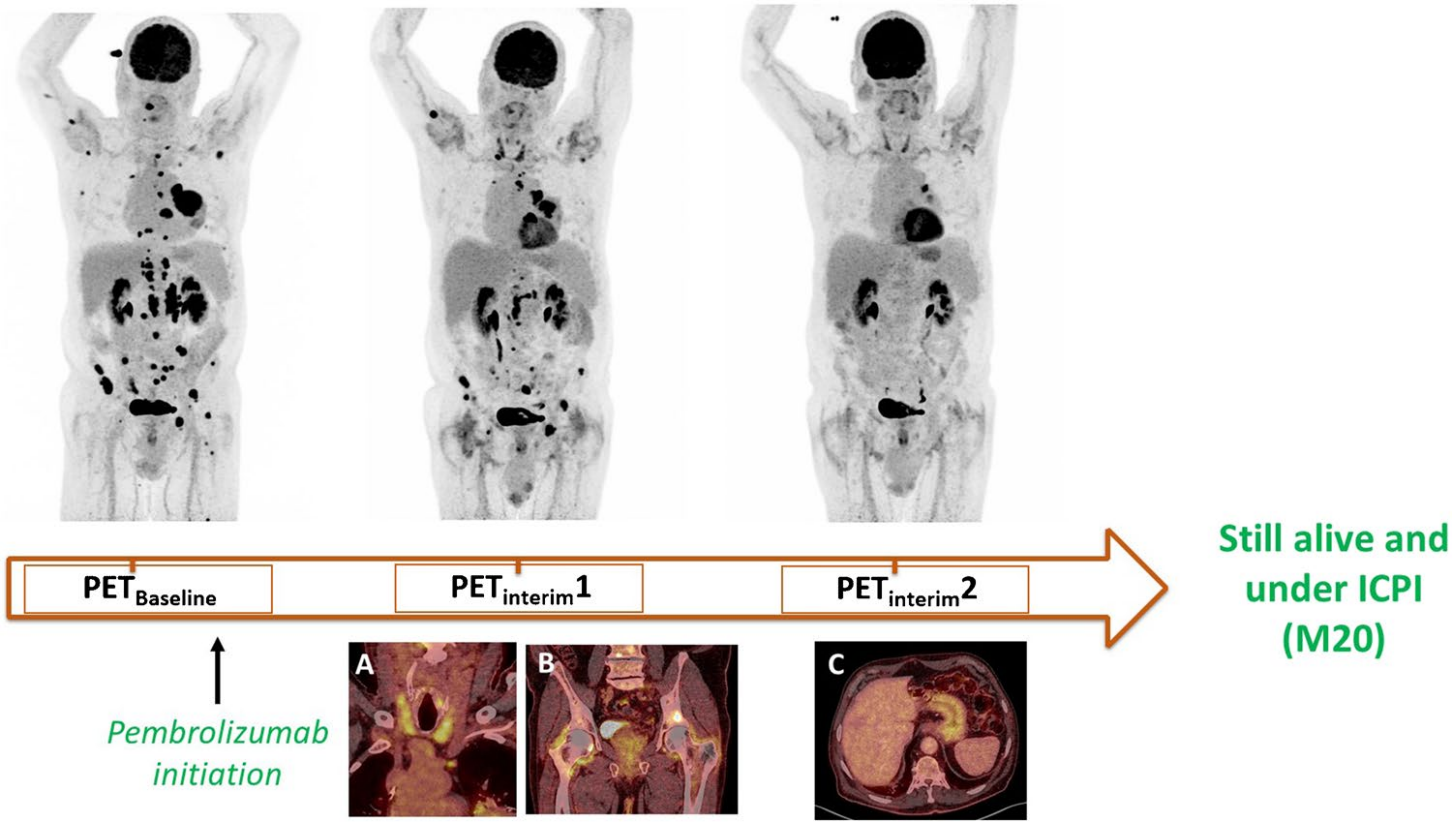
An 81-year-old man (from the exploratory cohort) treated with pembrolizumab and demonstrating a dissociated response on PET_Interim_1 (metabolic response of most lesions, but metabolic progression of a lesion on the right humerus head and of a right supra-clavicular lymph node) and a partial metabolic response on PET_Interim_2. On PET_Interim_1, immuno-induced thyroiditis (**A**) and immuno-induced-arthritis (pelvic and shoulder girdles) (**B**) occurred. On PET_Interim_2, an immuno-induced gastritis (**C**) was also observed. The patient was still benefiting from pembrolizumab 20 months after its initiation

Occurrence of ICPI-induced organ inflammation on ^18^FDG PET/CT (Table 2; Fig. 1):

**Table 2:** Occurrence of ICPI-induced organ inflammation on interim ^18^FDG PET

Immunotherapy-induced inflammation on PET_Interim_1 and/or PET_Interim_2	^Exploratory cohort, Nice^	^Validation cohort, Genova^
All organs		
Yes	67 (72.8)	39 (86.7)
No	25 (27.2)	6 (13.3)
Pneumonitis		
Yes	16 (17.4)	9 (20.0)
No	76 (82.6)	36 (80.0)
Thyroiditis		
Yes	17 (18.5)	4 (8.9)
No	75 (81.5)	42 (91.1)
Gastritis		
Yes	20 (21.7)	9 (20.0)
No	72 (78.3)	36 (80.0)
Midgut/hindgut inflammation		
Yes	31 (33.7)	18 (40.0)
No	61 (66.3)	27 (60.0)
Other organs’ inflammation		
Yes	9 (9.8)	2 (4.4)
No	83 (90.2)	43 (95.6)

Other organ: pancreatitis, ENT inflammation, osteo-articular inflammation, esophagitis, mesenteric panniculitis, mediastinal granulomatosis occurrence, pleuritis

The occurrence of at least one site of organ inflammation after introduction of ICPI was observed on PET_Interim_1 and/or PET_Interim_2 in 72.8% of patients (67/92).

The types of ICPI-induced organ inflammation observed were as follows:

- Midgut/hindgut inflammation in 33.7% (31/92) of patients.
- Gastritis (pathological uptake of the stomach and/or 1st duodenum) in 21.7% (20/92) of patients.
- Thyroiditis in 18.5% (17/92) of patients.
- Pneumonitis in 17.4% (16/92) of patients.
- Other organ inflammation in 9.8% (9/92) of patients: pancreatitis, ENT inflammation, arthritis, esophagitis, mesenteric panniculitis, pleuritis.

#### ***Univariate analysis of the association between patients’ characteristics and outcome (Table ******3******)***

**Table 3:** Univariate analysis of prognostic factors for PFS and OS (exploratory cohort)

	Progression-free survival				Overall survival			
	Events	HR	95%CI	*p* value*	Events	HR	95%CI	*p* value*
Age (years)								
< 65	27/44	1			25/44	1		
≥ 65	24/48	0.67	[0.38–1.2]	0.15	20/48	0.67	[0.37–1.2]	0.18
Sex								
Women	19/33	1			17/33	1		
Men	32/59	0.69	[0.39–1.2]	0.20	28/59	0.85	[0.46–1.6]	0.60
ECOG performance status								
0	11/24	1			9/24	1		
1	33/58	1.6	[0.77–3.2]		29/58	1.8	[0.81–3.9]	
2	7/10	2.2	[0.83–5.8]	0.25	6/10	3.3	[1.1–10]	0.08
Tumor histology								
Adenocarcinoma	38/72	1			33/72	1		
Squamous cell carcinoma	9/16	1.1	[0.54–2.3]	0.75	8/16	1.3	[0.59–2.8]	0.53
Current or former smoker								
No	12/18	1			11/18	1		
Yes	22/45	0.6	[0.3–1.2]	0.15	22/45	1.1	[0.52–2.3]	0.80
Previous chemotherapy								
No	12/24	1			11/24	1	[0.45–1.8]	
Yes	39/68	1.2	[0.61–2.2]	0.65	34/68	0.89	[0.45–1.8]	0.75
PD-L1 tumor expression								
0%	5/10	1			4/10	1		
1–49%	13/21	0.52	[0.18–1.5]		12/21	1	[0.33–3.2]	
≥ 50%	22/42	0.39	[0.14–1.1]	0.17	21/42	0.89	[0.3–2.6]	0.90
Treatment								
Pembrolizumab	28/50	1			27/50			
Nivolumab	22/39	1	[0.58–1.8]	0.86	18/39	0.65	[0.35–1.2]	0.17
iPERCIST (PET_Interim_1)								
CMR/PMR/SMD	8/33	1			7/33	1		
uPMD	43/59	5.5	[2.6–12]	< 0.0001	38/59	4.6	[2.1–10.0]	< 0.0001
Inflammation on PET_Interim_1/2 (all organs)								
No	14/25	1			13/25	1		
Yes	37/67	1.1	[0.59–2.0]	0.79	32/67	0.92	[0.48–1.8]	0.79
Immuno-induced gastritis on PET_Interim_1/2								
No	41/72	1			39/72	1		
Yes	10/20	1.5	[0.74–2.19]	0.13	6/20	2.6	[1.1–6.1]	0.026

^*^Log-rank test

*HR*, hazard ratio; *CI*, confidence interval

Association with PFS:

- Clinico-biological variables

None of clinical characteristic assessed (age, sex, ECOG performance status, tumor histology, smoking status, history of lung surgery/radiotherapy/chemotherapy, PD-L1 tumor expression, ICPI drug administered) was significantly associated with PFS, including PD-L1 tumor expression.

- PET imaging variables

Tumor metabolic response, using iPERCIST criteria on PET_Interim_ 1 and 2, was significantly associated with improved PFS (*p* < 0.001 for both analyses).

Occurrence of ICPI-induced organ inflammation on PET_Interim_1 and/or PET_Interim_2 was not significantly associated with PFS, either on a global or a site-per-site analysis.

Association with OS:

- Clinico-biological variables

Among the clinical and biological patients’ characteristics assessed (age, sex, ECOG performance status, tumor histology, smoking status, history of lung surgery/radiotherapy/chemotherapy, PD-L1 tumor expression, ICPI drug administered), none was significantly associated with OS.

Patients with a higher ECOG performance status tended to have a worse OS but this was not statistically significant (*p* = 0.08).

**Fig. 2:**
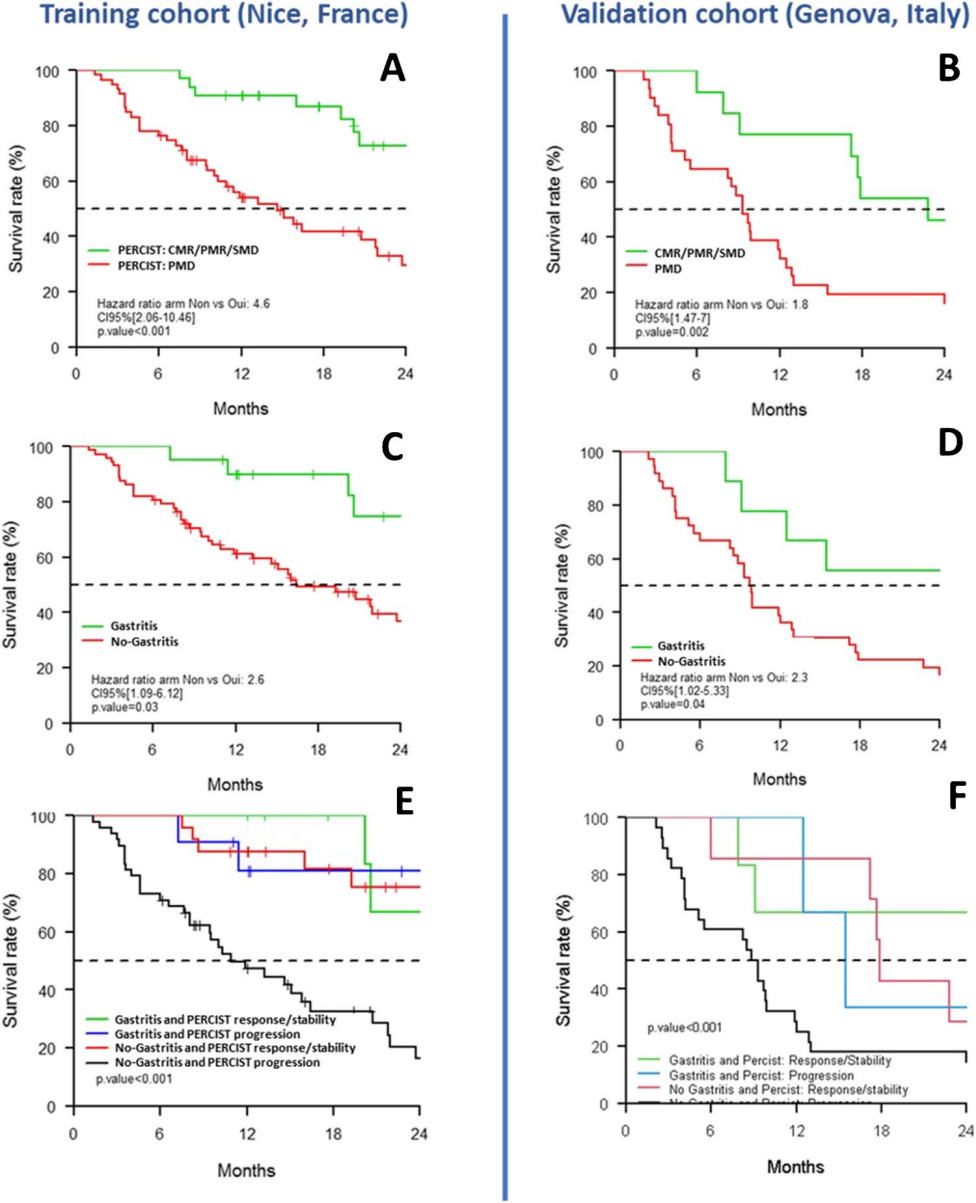
Patients’ overall survival curves according to iPERCIST criteria and immunotherapy-induced gastritis assessed on interim PET. Overall survival according to iPERCIST criteria on PET_Interim_1 in the exploratory (**A**) and validation (**B**) cohorts; the occurrence of immunotherapy-induced gastritis on PET_Interim_1/2 in the exploratory (**C**) and validation (**D**) cohorts; the combination of iPERCIST criteria and immunotherapy-induced gastritis in the exploratory (**E**) and validation (**F**) cohorts. CMR, complete metabolic response; PMR, partial metabolic response; SMD, stable metabolic disease; PMD, progressive metabolic disease

- Imaging variables (Fig. 2)

Tumor metabolic response, using iPERCIST criteria on PET_Interim_ 1 and 2, was significantly associated with improved OS (*p* < 0.001 for both analyses). Patients with a tumor response/stability on PET_Interim_1 had a 2.5-fold improved OS compared to patients with tumor progression (2-year OS = 73% vs 29%).

Immunotherapy-induced gastritis on PET_Interim_1 and/or PET_Interim_2 was significantly associated with improved OS (*p* = 0.026). Patients with early gastritis occurrence had a twofold improved OS compared to patients without it (2-year OS = 75% vs 37%). The other sites of ICPI-induced inflammation (i.e., pneumonitis, thyroiditis, etc.) were not significantly associated with OS.

Combining iPERCIST criteria and immunotherapy-induced gastritis occurrence in a sub-group analysis, the 2-year OS rate was significantly different between the 4 sub-groups of patients (*p* < 0.001).

#### Multivariate predictive model of overall survival

**Fig. 3:**
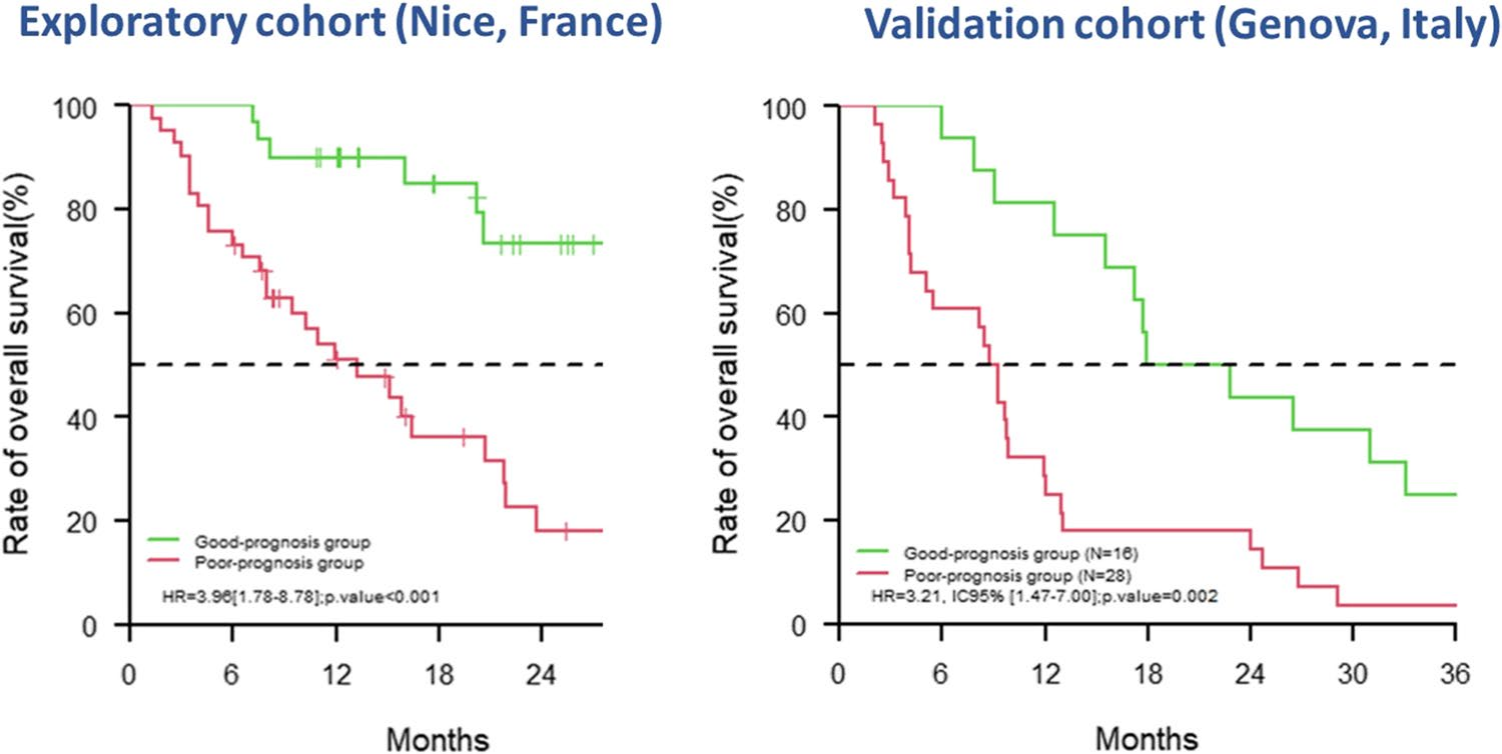
Patients’ overall survival curves according to the multivariate predictive model

The final model to predict 2-year OS, including iPERCIST tumor response (on PET_Interim_1) and immune-induced gastritis (on PET_Interim_1/2) as two complementary variables (Supplementary Table S2), had an area under the ROC curve of 72.7% (CI 95% = 57–88). According to ROC curve analysis, a score of − 0.19 was the optimal threshold for predicting patients’ OS at 2 years, with a sensitivity of 80.3% and a specificity of 69.2%. With this threshold, the median overall survival was 13.2 months (CI 95% = 8.0–20.7) in the poor prognosis group and was 29.7 months (CI 95% = 20.6–not reached) in the better prognosis group (*p* < 0.001) (Fig. 3).

### Validation cohort (Genova, Italy)

Forty-nine patients were prospectively included. Four patients were excluded because of PET_Interim_1 cancelation or wrong timing. Forty-five remaining patients were evaluated in this study. Patients’ characteristics are indicated in Table 1. All patients were treated with nivolumab, the two-thirds being treated in the 3rd or later line of treatment (Table 1).

Mean delay between baseline PET and initiation of immunotherapy was 17.1 ± 13.3 days. Mean time between introduction of immunotherapy and PET_Interim_1 was 52.7 ± 7.9 days. Mean time between introduction of immunotherapy and PET_Interim_2 was 98.3 ± 19.0 days.

Patients’ median follow-up was 56.6 months (CI 95% = 55.2–NA); Patients’ median progression-free survival was 5.9 months (CI 95% = 3.2–8.7) (Supplementary Fig. S1). Patients’ median overall survival was 9.9 months (CI 95% = 9.1–17.7) (Supplementary Fig. S2). The occurrence of at least one site of ICPI-induced organ inflammation was observed on PET_Interim_1 or PET_Interim_2 in 86.7% of patients (39/45).

The types of ICPI-induced organ inflammation observed on PET/CT are described in Table 2. ICPI-induced gastritis was assessed in 20.0% (9/45) of patients.

As observed in the exploratory cohort from Nice, iPERCIST tumor response on PET_Interim_1 was significantly associated with better OS (Fig. 2). Patients with a tumor response/stability had a 2.9-fold improved OS compared to patients with tumor progression (2-year OS = 46% vs 16%, *p* = 0.002).

Patients with an immuno-induced gastritis occurrence after ICPI initiation on PET_Interim_1 and/or PET_Interim_2 had a 2.7-fold improved OS compared to patients without it (2-year OS = 56% vs 17%, *p* = 0.04) (Fig. 2).

The other sites of organ inflammatory impairment (i.e., pneumonitis, thyroiditis, midgut/hindgut, other organs’ inflammation) were not significantly associated with OS.

Finally, the prognostic model obtained in the cohort from Nice, including iPERCIST response and immune-induced gastritis criteria, was assessed in this independent validation cohort, using the optimal threshold (= − 0.19) defined in the exploratory cohort. The AUC of ROC curve to predict 2-year OS was equal to 70.7 (CI 95% = 78.1–88.3) with a sensitivity of 72.0% and a specificity of 63.6%.

Having a longer follow-up in this validation cohort, we also assessed the prognostic value of the same model to predict 3-year OS: the AUC was equal to 78.2 (CI 95% = 57.1–99.2) with a sensitivity of 69.2% and a specificity of 80.0%.

The OS curves, distinguishing poor and good prognostic groups using this model, are given in Fig. 3.

## Discussion

Tumors and activated inflammatory cells both have increased expression of glucose transporters and glycolysis, leading to increased ^18^FDG uptake. Assessment of adverse inflammatory events following the introduction of ICPIs on ^18^FDG PET/CT imaging may precede clinical symptoms, potentially leading to the earlier therapeutic management of irAEs. However, the utility of ^18^FDG PET/CT in the follow-up of patients, particularly for the detection of inflammatory adverse events, has been poorly investigated up to now. In a recent study including patients with non-metastatic bladder cancer treated with neoadjuvant pembrolizumab, ^18^FDG PET/CT showed ICPI-induced organ inflammation in 39% of patients, corresponding to grade 1 or 2 clinically detectable irAEs in 45% of cases [21]. Mekki et al. demonstrated on a retrospective cohort (*n* = 53) wherein ^18^FDG PET/CT detected 74% of irAE in patients treated with anti-PD1 [22].

In our study, ^18^FDG PET/CT detects many ICPI-induced organ inflammation occurring during the first 3 months after ICPI initiation (72.8% of patients in the exploratory cohort and 86.7% in the validation cohort). This rate of inflammatory FDG uptake, either symptomatic or not, is very high, but in accordance with the also high incidence of irAE occurrence, described in a recent meta-analysis [23]. In NSCLC, the most common irAEs in patients receiving PD-1 therapies are endocrinopathies (mostly thyroiditis), pneumonitis, hepatitis, diarrhea/nausea, and arthralgia [1, 24, 25].

However, direct comparisons between the rates and types of clinical irAEs and inflammatory tissue uptake detected on PET cannot be done. Firstly, ICPI-induced organ inflammation assessed on ^18^FDG PET is not necessarily symptomatic. Secondly, only irAEs managed with specific treatments are usually reported in clinical trials, without considering irAE of grade 1. Lastly, some irAEs are clinically hard to diagnose due to their pleomorphic presentations, such as asthenia and myalgia [26].

### Predictive and prognostic values of irAEs during ICPI

Thus far, research on predictive biomarkers of ICPI efficacy has focused on tumor or micro-environmental biological parameters. Clinical biomarkers have been less studied. Previous studies have suggested that the occurrence of symptomatic irAEs is associated with a more favorable outcome to PD-1 inhibitors, probably due to cross-reactivity between tumor and host tissues [11, 27–31]. For example, a pooled multi-institutional cohort recently found that the incidence of grade ≥ 2 irAEs was associated with improved long-term survival across different malignancies treated with ICPI monotherapy [27]. This overall finding was confirmed in patients with lung cancer treated with an anti-PD(L)1 therapy. In another retrospective study including 270 patients with NSCLC receiving ICPIs in different lines, OS and PFS were significantly better for patients with reported irAEs than those without [28]. Outcome was not significantly different according to the different irAE grades. On a per-organ statistical analysis, thyroid dysfunction was the only specific irAE positively associated with improved OS and PFS.

The prognostic value of immune-induced inflammatory organ uptake assessed with ^18^FDG PET has been less investigated than clinical irAEs. As a previous study conducted on a small cohort of 25 patients with metastatic melanoma [32], we did not found a global prognostic value of immune-induced organ inflammation on ^18^FDG PET/CT performed in the early patients’ follow-up. But on a per-organ analysis, the occurrence of a gastritis during the first 3 months of ICPI treatment was significantly associated with improved OS (but not PFS) in the exploratory cohort. This discrepancy between OS and PFS is common when evaluating immunotherapy and may be explained by the difficulty of determining a reliable PFS in that setting. Patients with an immune-induced gastritis had a twofold improved 2-year OS compared to patients without it. This result was confirmed in the external validation cohort, in a different hospital and using a different PET/CT system. This independent validation was important to avoid false biomarker discovery and confirm that immuno-induced gastritis on PET is a reproducible predictive biomarker of improved clinical outcome. Moreover, combining tumor response (iPERCIST criteria) and gastritis occurrence can more accurately predict patient’s overall survival, as demonstrated by the predictive model. This model could identify long-term survivors with immunotherapy in advanced NSCLC (3-year OS) with a 69% sensitivity and 80% specificity in the external validation cohort. From a clinical perspective, ICPI-induced gastritis could be an additional argument for ICPI efficacy in case of doubtful or atypical response patterns on ^18^FDG PET/CT, such as dissociated response or pseudo-progression, often observed in the monitoring of patients treated with ICPI [14, 33].

### Previous knowledge on immunotherapy-induced gastritis

Because gastritis symptoms can be insidious, overlapping with the common symptoms of metastatic cancer, immuno-induced gastritis may frequently be under-diagnosed without endoscopic confirmation [34]. The current literature is mostly comprised of case reports and series of patients [34–36]. Only one large retrospective study (*n* = 205) has described the incidence of ICPI-gastritis at 5.4% of patients receiving immunotherapy for melanoma [37].

The occurrence of diffuse uptake of the gastric wall during ICPI treatment, related to immune-induced gastritis, has been mentioned in few previous ^18^FDG PET/CT case reports [21, 32, 38]. In the neoadjuvant setting of bladder cancer, Marandino et al. noted diffuse stomach uptake in 13.6% of patients (14/103) [21]. In this case, gastritis was always asymptomatic and therefore not confirmed by a gastroscopy.

There is currently no clear physio-pathological explanation of the association between immune-induced gastritis and improved outcome. The patient’s microbiota may be an interesting first track to explore this association. Indeed, the role of microbiota in regulating response and toxicities to ICPIs in cancer is increasingly recognized [39, 40], as the gut microbiome can create a pro- or an anti-inflammatory environment. Certain bacterial strains, such as *Faecalibacterium*, confer sensitivity to ICPI, also increasing the risk of irAEs [40], whereas dysbiosis or loss of microbial diversity is associated with poor response to ICPIs [41]. Recent studies have shown that the stomach also harbors a specific and diverse microbiota, although different from the rest of the digestive tract [42]. Interestingly, *Helicobacter pylori* (HP) can induce PD-L1 expression on gastric epithelial cells [43] and, in a large pooled analysis including 1512 patients with NSCLC randomly receiving atezolizumab or docetaxel, treatment with proton pump inhibitors (PPIs) was demonstrated to be specifically associated with a poorer outcome in patients treated with ICPIs [44]. Similar results were found in another retrospective study [45]. These data suggest that PPIs that induce gastric pH change may lead to subsequent alteration of gastric microbiota and influence the systemic tumor response to ICPIs. Mechanistic studies into the microbiome and gastric immunological pathways are needed to elucidate these physio-pathological mechanisms.

Despite the well-described role of the gut microbiome (assessed from stool samples) in shaping systemic immune responses, the occurrence of ICPI-induced midgut/hindgut inflammation on ^18^FDG PET/CT was not significantly associated with patient’s outcome. One explanation may be that we collected the occurrence of ICPI-induced organ inflammation only in a binary manner (occurrence versus nonoccurrence), without quantifying the magnitude of the intensity and extent of inflammatory uptake assessed on PET_Interim_1/2. It could have been relevant to quantify the severity of ^18^FDG uptake to distinguish patients with minor and major inflammatory involvement of the midgut/hindgut. This quantitative work could be the subject of a future specific ancillary study.

### Limitations of the study

This study has some limitations. Firstly, although most irAEs occur within the first 3 months of treatment, the 3-month follow-up may be too short to detect some types of delayed irAEs such as nephritis [24, 27]. Secondly, we did not make any correlation between ICPI-induced tissue inflammation observed on PET and the occurrence of clinically symptomatic irAEs, as this was not the main objective of this observational study. Thirdly, we focused only on patients treated with ICPI as single agent therapies. Other studies should evaluate the impact of irAEs on clinical outcomes in patients treated with a combination of PD-L1 inhibitors and chemotherapy, the new first-line gold standard for many patients. However, the relationship between irAEs and IPCI efficacy will be more challenging to assess in combination treatment strategies.

Finally, significant differences in survival were observed between the exploratory and validation cohorts. This may be explained by differences in the clinical characteristics of the two cohorts. Patients from Nice mainly received ICPI in the first and second lines, whereas two-thirds of Genova patients were treated in second or later lines. Although the early objective response rate to ICPI did not differ significantly between the 2 cohorts (29.3% vs 27.3% on PET_Interim_1), tumor escape and progression occurred earlier in the more heavily treated cohort, prior to ICPI, with a median PFS of 14.3 months (Nice) vs 5.9 months (Genova).

## Conclusion

Beyond the standard assessment of metastatic lung cancer response to ICPIs, ^18^FDG PET/CT can also lead to early detection of various organ inflammatory events occurring during treatment, either symptomatic or not. Among them, immuno-induced gastritis, revealed early by interim ^18^FDG PET in around 20% of patients with NSCLC, is a novel and strong imaging biomarker of improved overall survival, providing additional prognostic value to (i)PERCIST criteria. Regarding the clinical relevance of this finding, ICPI-induced gastritis could be an additional argument for ICPI efficacy in case of doubtful or atypical response patterns on ^18^FDG PET/CT, such as dissociated response or pseudo-progression.

## Supplementary Information

Below is the link to the electronic supplementary material.

Supplementary file1 (DOCX 1875 KB)
