# Supplementary material for: Prognostic value of immunotherapy-induced organ inflammation assessed on 18FDG PET in patients with metastatic non-small cell lung cancer
Source: Eur J Nucl Med Mol Imaging. 2022 May 14;49(11):3878–91. doi: 10.1007/s00259-022-05788-8 (PMC9399195; doi:10.1007/s00259-022-05788-8)
Supplement: Supplementary file 1 — Supplementary file1 (DOCX 1875 KB) [file 259_2022_5788_MOESM1_ESM.docx]

**Supplementary Table S1: Summary of PERCIST and iPERCIST criteria definitions**

|  | **PERCIST** (23) | **iPERCIST** (18,20) |
| --- | --- | --- |
| **CMR** | Complete disparition of ^18^FDG uptake of all lesions | Complete disparition of ^18^FDG uptake of all lesions |
| **PMR** | ≥ 30% decrease in the target tumor SUL_peak_ | ≥ 30% decrease in the target tumor SUL_peak_ |
| **SMD** | Neither CMR, PMR nor PMD | Neither CMR, PMR, uPMD or cPMD. |
| **PMD** | ≥ 30% increase in SUL_peak_ or advent of new FDG avid lesion(s) | ≥ 30% increase in FDG SUL_peak_ or advent of new ^18^FDG-avid lesion(s). This first progression is termed **uPMD.**  **uPMD** needs to be confirmed by a second metabolic progression of lesions on a new PET exam performed 4–8 weeks later and is then termed **cPMD**;  If uPMD is followed by PMR or SMD, the bar is then reset and the episod is ex-post considered as a pseudo-progression.  Clinical status is considered when deciding whether treatment should be continued after UPMD. |

**CMR**: Complete metabolic response; **PMR**: Partial metabolic response; **SMD**: Stable metabolic disease; **PMD**: Progressive metabolic disease; **uPMD**: Unconfirmed progressive metabolic disease; **cPMD**: Confirmed progressive metabolic disease; SUL: Standardized uptake value, normalized to the lean body mass; PERCIST: Positron emission tomography response criteria in solid tumors; iPERCIST: Immune positron emission tomography Response Criteria in solid tumors.

**Supplementary Table S2: Results from multivariate cox regression model containing all explanatory variables (full model) predicting likelihood of overall survival.**

| **Term** | **β** | **exp (β)** | **95% CI for exp (β)** | | **se(β)** |
| --- | --- | --- | --- | --- | --- |
|  |  |  | **lower** | **upper** |  |
| iPERCIST response (PET_interim_1) (β_1_ ) | 1.287 | 3.622 | 1.396 | 9.395 | 0.486 |
| immune-induced gastritis (β_2_ ) | 0.741 | 2.098 | 0.806 | 5.457 | 0.487 |

**Supplementary Figure S1. Patient’s progression free survival curves
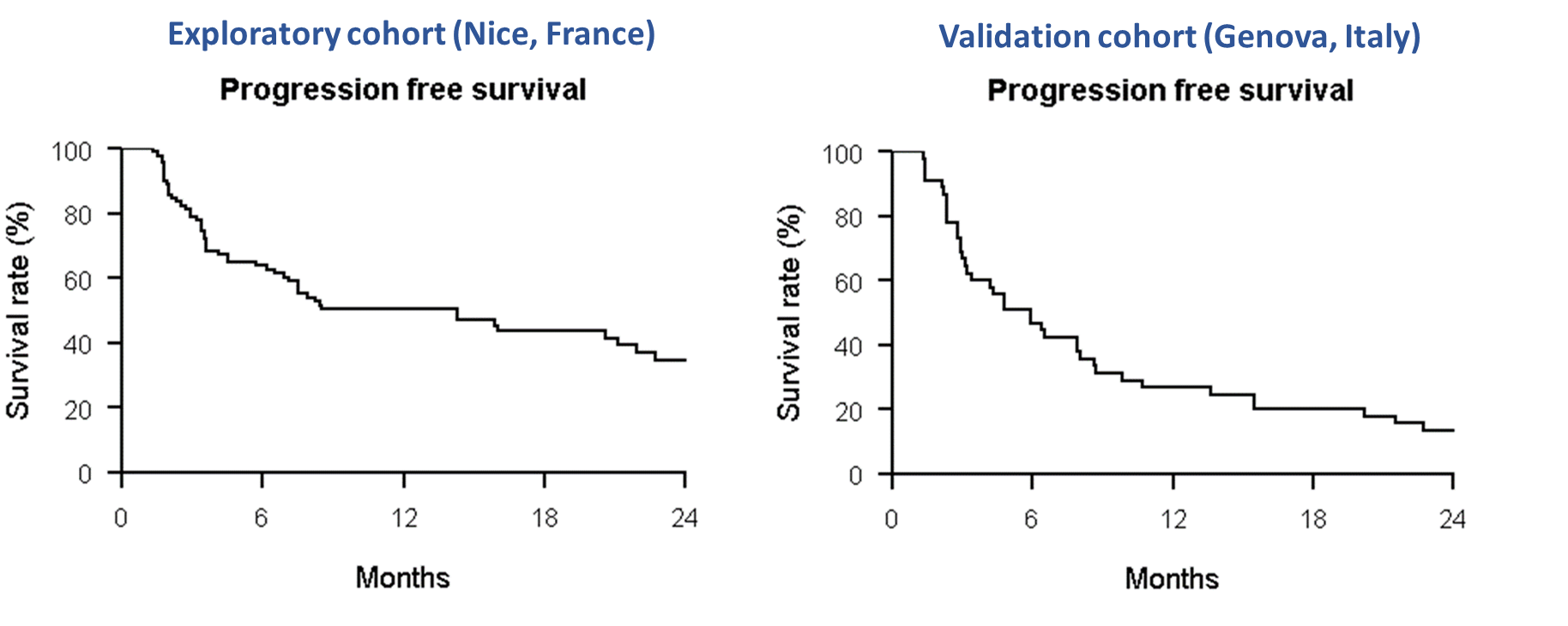
**

**Supplementary Figure S2. Patient’s overall survival curves**

**
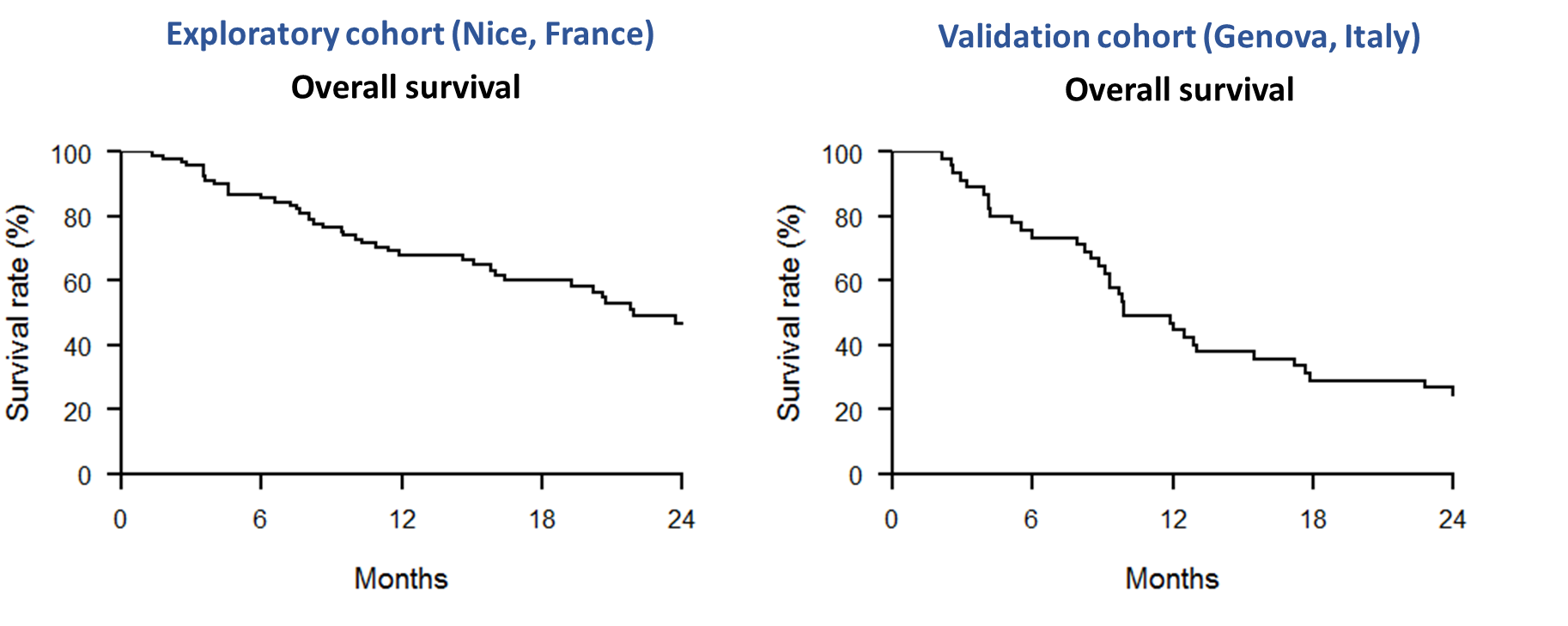
**

**Supplementary Figure S3. Patient’s overall survival curves according to the multivariate predictive model**
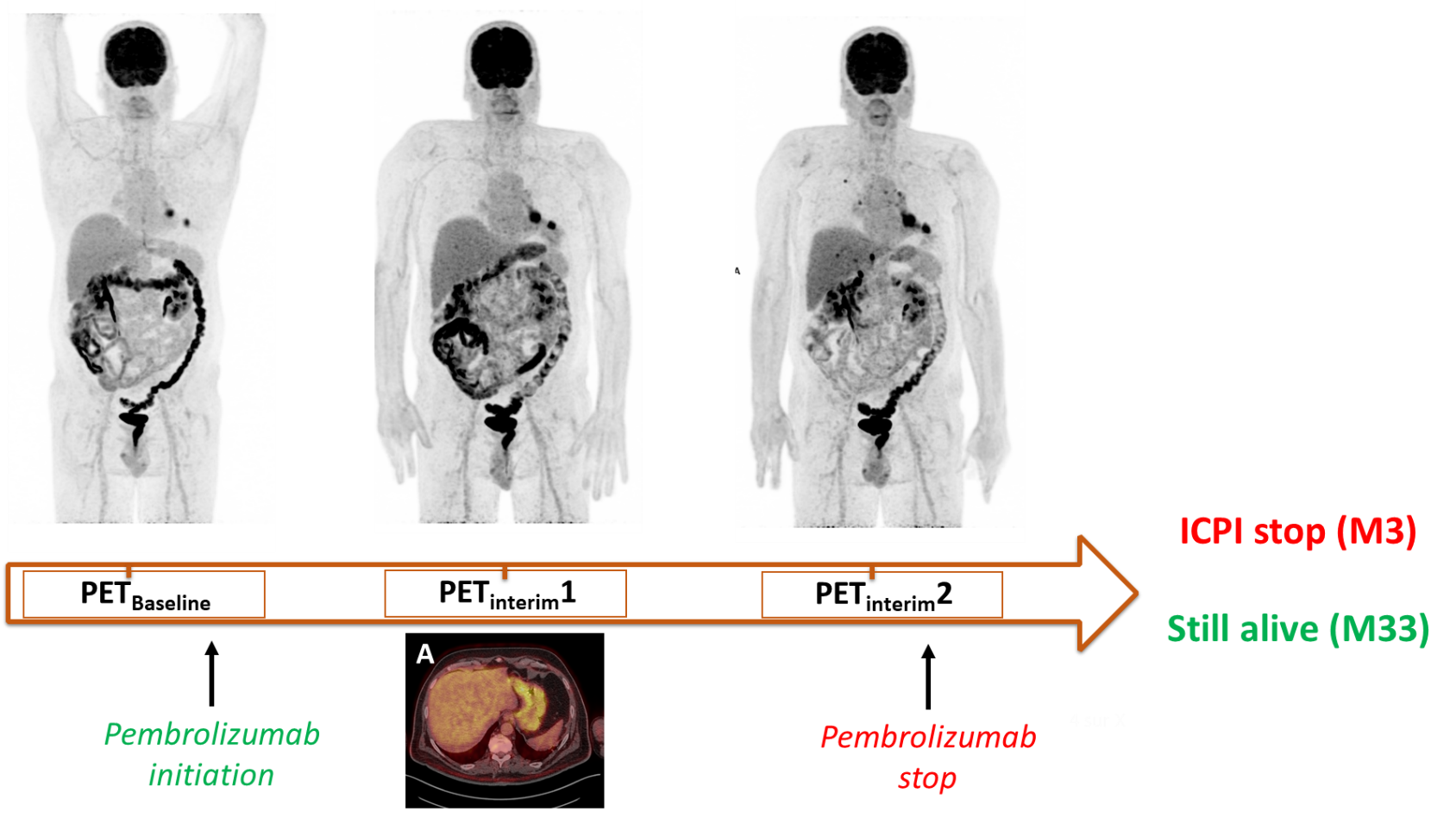


A 61-year-old man (from the exploratory cohort) treated with Nivolumab and demonstrating a progressive metabolic disease on PET_interim_1, confirmed on PET_interim_2.

On PET_interim_1, immuno-induced-gastritis (A) occurred and was still assessed on PET_interim_2 (also less intense). The patient stopped the ICPI due to this confirmed progressive metabolic disease and benefited from next-line chemotherapy. He was still alive 30 months later.

**Supplementary Figure S4.**
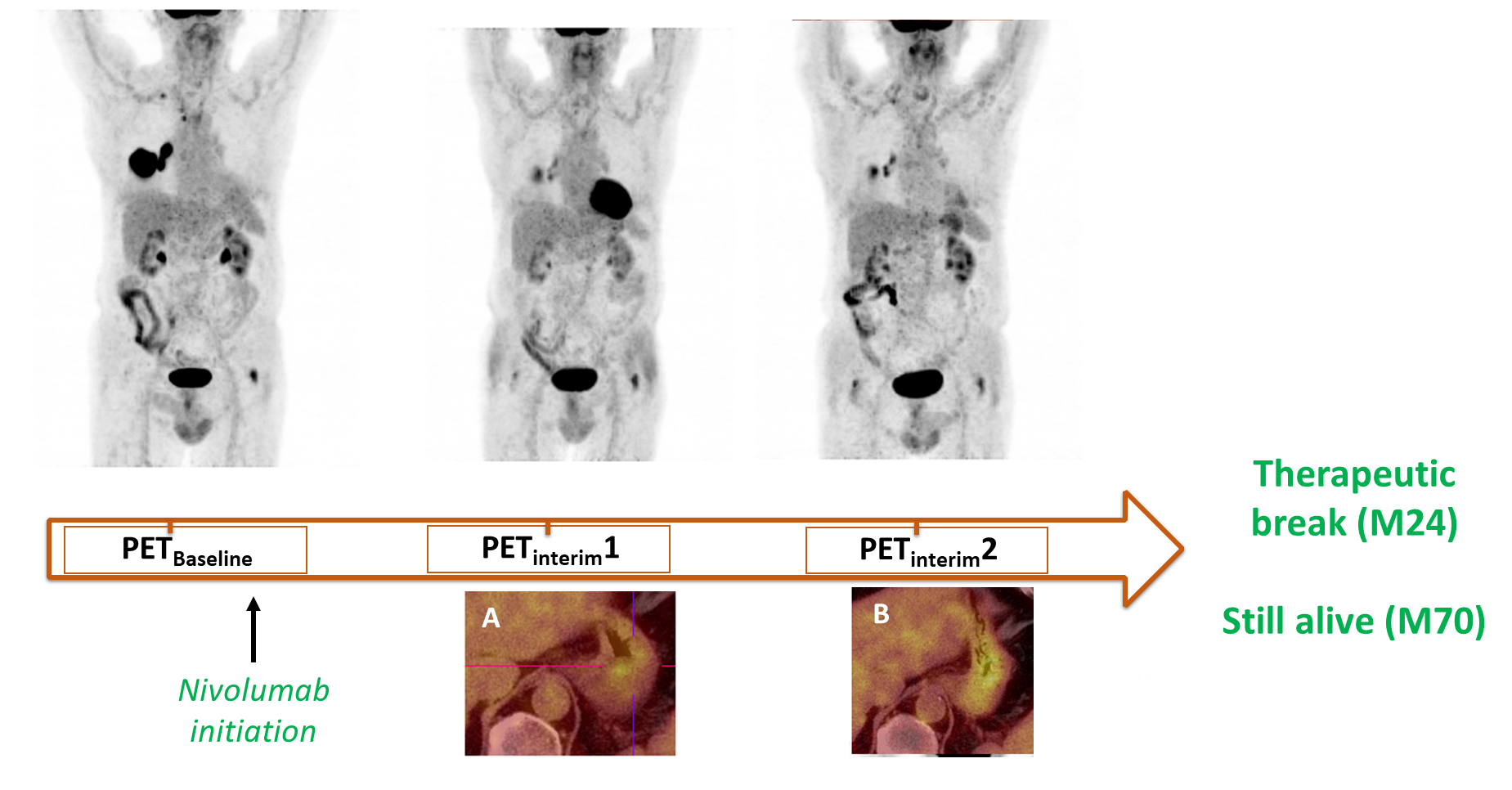


A 78 years old man (from the validation cohort) treated with nivolumab and demonstrating a partial metabolic response on PET_interim_1 and an unconfirmed progressive metabolic disease on PET_interim_2 (metabolic progression of a lung nodule, a right hilar and a right supraclavicular lymph node. A slight immuno-induced gastritis was observed on PET_interim_1 (A) and increased on PET_interim_2 (B). Because the clinical status was good, ICPI was maintained, and the patient finally benefited from a therapeutic break after 2 years of effective ICPI treatment. He was still alive 5 years after treatment initiation.
